# Clisagri: An R package for agro-climate services

**DOI:** 10.1016/j.cliser.2020.100197

**Published:** 2020-12

**Authors:** A. Ceglar, A. Toreti, M. Zampieri, V. Manstretta, T. Bettati, M. Bratu

**Affiliations:** a European Commission, Joint Research Centre, via Enrico Fermi 2749, 21027 Ispra, Italy; b HORTA, Via Egidio Gorra 55, 29122 Piacenza, Italy

**Keywords:** Climate service, Decision support, Winter wheat, Drought, Heat stress, Seasonal prediction

## Abstract

Crop yields are affected by unfavourable/extreme weather and climate events occurring during sensitive growth stages. Understanding the risks associated with these events is essential to adapt agro-management decisions and reduce losses. For this purpose, we propose a targeted climate service integrating a dynamic crop phenology model with an approach based on dedicated agro-climate risk indicators. The initial set of these indicators has been developed in a co-design approach with agronomists and durum wheat farmers participating as end-users in the H2020-MedGOLD project. Four groups of indicators characterize drought events, excessive wetness, cold stress and heat stress during sensitive growth stages. The proposed approach has been fully implemented as an R-package named *Clisagri*. The package is complemented with a set of optimization functions, which target optimal variety selection in terms of crop cycle duration.

## Highlights

• A new climate service tool *Clisagri* has been developed for the agricultural sector.
• *Clisagri* models the risk of unfavourable climate events during crop growth.
• *Clisagri* quantifies drought, wetness, heat stress and cold stress exposure.
• Dynamic phenological model enables targeting sensitive growth stages.
• Optimization enables optimal variety selection in terms of crop cycle duration.

Practical implications Crop growth and development are crucially dependent on weather conditions preceding and occurring during the growing season. Crop yield quantity and quality are negatively affected by unfavourable/extreme weather and climate events, such as: heat stress, drought, excessive rainfall and frost kill. Due to current and projected climate changes, it has become increasingly important for farmers to raise their preparedness to these harmful events. In this context, sectorial climate services can effectively support and inform agricultural decisions, making the agricultural production more resilient, sustainable and efficient.

This study introduces an innovative co-designed agro-climate service tool *Clisagri* that translates state-of-the-art climate data and predictions at seasonal time scales, and beyond, into valuable information for a wide range of end users in the agricultural sector. *Clisagri* has been co-designed with farmers and agronomists, who have characterized weather and climate events during the wheat growing season potentially resulting in: losses of crop yield quality and quantity, occurrence of diseases and weeds, problems related to grain storage after harvest and difficulties related to crop fertilization. During the co-design approach we translated their perceived harmful weather and climate events into a set of agro-climate indicators. It is worth to highlight that for each identified event, farmers participating in this co-design exercise outlined measures and actions to reduce the expected impacts. Nevertheless, an intermediate agent may be necessary in some cases to convert the output of *Clisagri* into a clear operative set of recommendations for farmers in the field. Therefore, *Clisagri* has been already integrated into a Decision Support System (DSS) developed by HORTA (https://www.horta-srl.it), a permanent platform for enhancing results from research in the agro-alimentary sector. The HORTA DSS reaches out hundreds of Italian farmers and beyond, that are already experimenting the added value *Clisagri*.

*Clisagri* is based on a set of dynamic agro-climate indicators that bring key information on climate related risks during different stages of the crop growth. A dynamic approach has been implemented to assess the risks associated with unfavourable/extreme weather and climate events during sensitive crop phenological stages by integrating dedicated indicators with a dynamic model to predict crop phenology. *Clisagri* quantifies the occurrence of different weather and climate events (including extremes) such as: drought, excessive wetness, heat stress and cold stress during sensitive crop growth stages. These sensitive stages generally occur in different periods every year as a consequence of inter-annual climate variability, and *Clisagri* offers an effective way to dynamically take this variability into account.

Furthermore, *Clisagri* is designed to test a complete range of possible crop varieties, and integrates an optimization process based on genetic algorithm to select the variety that is the ‘best fit’ for given climatic conditions. The latter can be reflecting the observed climate conditions as well as climate predictions and projections, ranging from seasonal to multi-decadal time scales. Indeed, different time scales can provide useful information for a range of farm activities as well as on crop breeding.

The paper, which is structured around the functionality of *Clisagri*, describes its key features and practical implications on a case study focused on durum wheat production in the Mediterranean environment. Possible future extensions are also discussed.

## 1 Introduction

Global agricultural production has been increasingly exposed to unfavourable/extreme weather and climate events, with future climate change projected to exacerbate in terms of frequency, severity, and spatial extent of extreme events (Ceglar et al., 2019a, Zampieri et al., 2019, Zhu and Troy, 2018). A number of stress factors affects crop growth, including heat stress, drought stress, excessive rainfall (water logging and water lodging) and frost kill (e.g. Toreti et al., 2019a, Trnka et al., 2014, Zampieri et al., 2017).

The increasing risk of crop failures due to biotic and abiotic factors points to the need of joint modelling and experimental efforts to better quantify and then predict the effects of these events during sensitive crop development stages such as flowering and harvest (Porter and Semenov, 2005). The development of sectoral climate services to support and inform agricultural decisions (and become a key component of adaptation strategies) is essential to build more resilient, efficient and sustainable agriculture (Falloon et al., 2018).

Crop models represent an indispensable tool in agricultural decision processes (Jones et al., 2017) as they integrate the combined effect of agro-management practices and climate conditions into informative indicators such as crop yield at the end of growing season. The application of crop models to inform the agricultural decision process usually requires a carefully designed sensitivity analysis and calibration, which brings the model output closer to the observed crop related variables (e.g. crop yield) from field experiments (Wallach et al., 2018).

On the other hand, indicator-based approaches represent a valuable alternative to assess the risk when it comes to the impacts of unfavourable/extreme weather and climate events on crop growth (e.g. Caubel et al., 2015, Caubel et al., 2017, Trnka et al., 2014). This approach can provide clear and simplified, yet robust, representation of crop exposure along the entire growing season. It is especially convenient for climate change studies as it provides simple and synthetic information that can be used by stakeholders at all levels (Caubel et al., 2015). Indicator-based frameworks can also support the identification of climate-related limitations to crop yield potential by estimating the likely impact on agricultural suitability zones (Caubel et al., 2017, Holzkämper et al., 2013, Holzkämper et al., 2011) as well as climate risks associated with projected climate conditions (Caubel et al., 2015).

Climate impact assessments dealing with risks in agricultural production are usually addressing the longer time scales, ranging from several decades up to a century ahead. While, the shorter time scales, from the next season to the next 5–10 years, are still largely unexplored. Here we aim to bridge this gap by proposing a tool able to integrate observations as well as climate predictions and projections. For this purpose, we developed a dynamic approach to assess the risk associated with unfavourable/extreme weather and climate events occurring during sensitive crop phenological stages. We integrate a dynamic crop phenology model with a suite of dedicated agro-climate risk indicators. This complete tool has been implemented as an R package named *Clisagri.* While it can be applied to a wide range of agro-climatic conditions and crops, it is here presented in a case study for durum wheat production in the Mediterranean region.

## 2 Methods

### 2.1 Dynamic modelling approach

Winter wheat quantity and quality can be severely affected by unfavourable/extreme weather and climate events occurring during sensitive growth stages. For instance, heat stress and drought, occurring around the short period of flowering, can substantially reduce yield potential (e.g. Barnabás et al., 2008, Porter and Semenov, 2005).

Accurate simulation of the phenological development is therefore essential, and it is here provided by a dynamic model of crop phenology. The derived development stages are then used to calculate the agro-climate indicators (i.e. the indicators are linked to specific phenological stages).

#### 2.1.1 Phenological development – standard approach

The phenological model used here to simulate the crop development is based on the accumulation of daily effective temperature; the latter being calculated as the difference between daily average temperature and a base temperature, for winter wheat assumed to be 0 °C. In case this difference is negative, the effective temperature is set to 0 °C. This daily accumulation is named development rate. This rate remains constant above a certain maximum effective temperature (for winter wheat assumed to be 30 °C) and is corrected for the effects of vernalization and photoperiod (Ewert et al., 1996; Porter, 1993):

(1) $$\mathrm{DVS}=\left\{\begin{array}{c} \sum_{i}\frac{\max(0,min\left(\left(T_{i}-T_{b}\right),T_{\max,e}\right))}{TSUM1}∙V_{f,i}∙P_{f,i}\;for\;DVS\leq 1\\ \sum_{i}\frac{{\max(0,min((T}_{i}-T_{b}),T_{\max,e}))}{TSUM2}\;for\;1<DVS\leq 2 \end{array}\right)$$

where *DVS* represents the development stage (0 – emergence, 1 – flowering and 2 – maturity), *T_b_* is the base temperature, *T_i_* represents the daily average temperature, *T_max,e_* is the maximum effective daily average temperature, $V_{f,i}$ represents the vernalization factor and $P_{f,i}$ is the photoperiod factor on day *i*, and *TSUM1* and *TSUM2* represent the length (in effective degrees days) of vegetative and reproductive periods, respectively. Detailed description of this phenological model together with its calibration for winter wheat in Europe can be found in Ceglar et al. (2019b). This approach has been also implemented in the WOFOST crop growth model (de Wit et al., 2019).

**Fig. 1:**
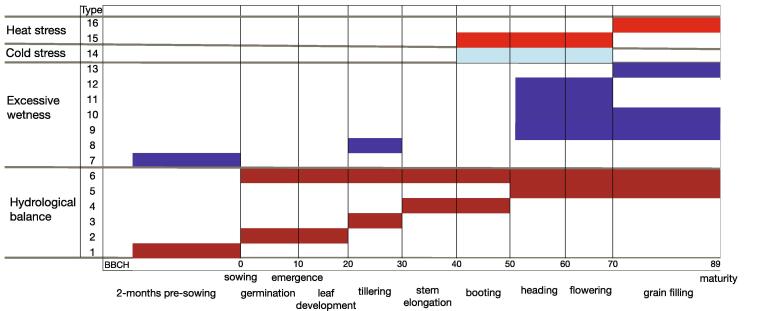
Different types of agro-meteorological indicators are needed to fully characterize meteorological conditions during different stages of winter wheat growth. Four groups of indicators are here chosen to characterize hydrological balance, excessive wetness, cold stress and heat stress conditions.

The output of this phenological model consists of daily *DVS* values, which can be linked to different crop growing stages, such as emergence, tillering, booting, heading, flowering and maturity (Fig. 1). The crop growing season can therefore be analysed in terms of phenological development, which allows to link the occurrence of unfavourable/extreme weather and climate events with specific crop development stages (phenological timing) rather than using fixed calendar period (Fig. 1). However, using the standard 2-phase approach we can only control the thermal requirements between sowing and flowering (*TSUM1*) and/or flowering and maturity (*TSUM2*). To control the duration of different sub-phases (such as booting-flowering), a multi-phase model must be introduced.

#### 2.1.2 Multi-phase model

Certain developmental phases, such as the period between terminal spikelet formation and flowering, are crucial for yield determination. It is therefore important for breeders to manipulate the duration of these phases to customize cultivars for specific environments (Reynolds et al., 2011). For instance, the breeding of durum wheat varieties in Spain has reduced the duration of the period between sowing and flowering (Isidro et al., 2011). However, in those varieties the sub-phases changed differently – a reduction has been observed for the period between sowing and terminal spikelet formation, while the duration of the period between booting and flowering has increased. To support variety selection based on breeding, which is targeting different sub-phases, a multi-phase model is made available in the *Clisagri* package.

The multi-phase model predicts the beginning and the end of six crop growth phases: PH1 – sowing to emergence, PH2 – emergence to tillering, PH3 – tillering to stem elongation, PH4 – stem elongation to booting, PH5 – booting to flowering, and PH6 – flowering to maturity (Rossi et al., 1997):

(2) $$\mathrm{DVS}=\left\{\begin{array}{c} \sum_{i}\frac{{\max(0,min((T}_{i}-T_{b1}),T_{\max,e}))}{{\mathrm{TSUM}}_{1}}\;for\;0<DVS\leq 1\\ \sum_{i}\frac{\max(0,min\left(\left(T_{i}-T_{b2\cdots b5}\right),T_{\max,e}\right))}{{\mathrm{TSUM}}_{2\cdots 5}}∙V_{f,i}∙P_{f,i}\;for\;1<DVS\leq 5\\ \sum_{i}\frac{{\max(0,min((T}_{i}-T_{b6}),T_{\max,e}))}{{\mathrm{TSUM}}_{6}}\;for\;5<DVS\leq 6 \end{array}\right)$$

where ${\mathrm{TSUM}}_{1\cdots 6}$ represent the thermal requirements and $T_{b1\cdots b6}$ the base temperature for each sub-phase. The photoperiod and vernalization factors are considered from PH2 to PH5. The *DVS* in this case ranges between 0 (sowing) and 6 (maturity).

The multi-phase approach should be a preferred choice to simulate phenological development to inform crop breeders who target specific sub-phases.

#### 2.1.3 Agro-climate conditions during sensitive growth stages

Agro-climate conditions during sensitive phenological stages are derived by using a set of indicators. An initial set of 16 indicators has been developed based on a co-design exercise involving Italian durum wheat farmers, done in the framework of the H2020-MedGOLD project. These indicators can be grouped into four categories: hydrological balance, excessive wetness, cold stress and heat stress (Fig. 1).

Hydrological balance indicators are used to estimate drought and/or wetness severity during different stages of crop growth (Table 1). For this purpose, the Standardized Precipitation-Evapotranspiration Index (SPEI, Vicente-Serrano et al., 2010), a multi-temporal-scale index quantifying persistent anomalies in soil water balance over different time periods, is used. The SPEI is based on climatic water balance, i.e. the difference between water availability (precipitation) and atmospheric water demand (reference evapotranspiration, referring to evapotranspiration above well-watered actively growing grass of uniform height, completely shading the ground). Reference evapotranspiration is estimated using the modified Hargreaves-Samani method (Droogers and Allen, 2002), which requires daily minimum and maximum temperatures, daily precipitation and location latitude as inputs (the latter is necessary to calculate extraterrestrial radiation). The ability of SPEI to capture well the impact of drought on European agricultural production has been shown in recent studies (Ceglar et al., 2018, Zampieri et al., 2017, Stagge et al., 2015). Here we have developed and implemented a non-parametric SPEI calculation procedure, which translates empirical cumulative distribution of climatic water balance into standardized water balance anomalies. The entire set of hydrological balance indicators in Table 1 is based on SPEI, calculated for different periods during the growing season. Hydrological balance indicators effectively support farmers on decisions related to: variety selection, sowing date, most suitable field operations, irrigation, application of fertilizers and optimal crop protection strategies (Table S1).

**Table 1:** Hydrological balance indicators characterizing conditions during different periods of winter wheat growth. All indicators are based on the Standardized Precipitation Evapotranspiration Index (SPEI).

Indicator	Phenological stage	Impacts on crop growth considered*
1	Pre-sowing	Drought or overwet conditions can influence field preparation activities, restricting the possibility of using appropriate sowing windows
2	Sowing - emergence	Drought can adversely impact seed germination, which can result in decreased germination rate, coleoptile length, seedling vigor, root length and shoot length. On the other hand, over-wet conditions lead to increased risk for disease occurrence.
3	Tillering	Drought limits the development of root system which can lead to decrease in: leaf area, leaf number per plant, leaf size and leaf longevity (Zhang et al., 2018, and reference therein). Contrarily, over-wet conditions lead to increased risk for disease occurrence.
4	Stem elongation – booting	Drought can lead to decreased plant height, number of grains per panicle or panicle number per plant or area (Zhang et al., 2018). Wetness increases the risk of diseases.
5	Heading – maturity	Drought can lead to reduced seed setting rate and reduce grain filling rate, leading to lower total biomass per plant at harvest. Over-wet conditions can cause severe reduction of yield and grain quality, increased harvest losses and exposure to diseases.
6	Sowing – maturity (entire season)	Drought can cause severe reduction of yield or crop die-back. Over-wet conditions can cause spread of diseases, severe reduction of growth or crop die back.

* Potential impacts on crop growth were collected from farmers feedback (MEDGOLD project), and literature review.

Excessive wetness indicators are based on counting the number of days with daily rainfall exceeding specified threshold and are, similar to hydrological balance indicators, calculated for different phenological stages. These indicators aim to capture the conditions that could lead to anoxia/hypoxia in the root zone, increasing the risk of diseases and impaired canopy structure caused by lodging (Table 2). The excessive wetness indicators can provide useful guidance for the selection of optimal crop protection strategy, application of optimal amount of fertilizers and planning of the most suitable grain storage strategy (Table S1).

**Table 2:** Excessive wetness indicators characterizing conditions during different periods of winter wheat growth.

Indicator	Phenological stages	Description	Impacts on crop growth considered
7	Pre-sowing	Rainfall cumulate	Delayed sowing and reduced sown areas
8	Tillering	Number of days with rainfall above 10 mm	Higher number of wet days can lead to soil saturation and increased exposure to diseases
9	Tillering	Number of days with heavy rain above 40 mm	Waterlogging can cause hypoxia or anoxia in the root zone, plants cannot absorb nitrogen from the soil, in the worst case tillering does not occur. Increased risk of diseases and reduced radiation use efficiency (Mäkinen et al., 2018, Trnka et al., 2014).
10	Heading-maturity	Number of days with rainfall above 5 mm	Higher number of wet days can lead to soil saturation and increased exposure to diseases
11	Heading – maturity	Number of days with rainfall above 40 mm	Lodging destroys canopy structure, it can cause severe reduction of yield and of grain quality, through increased harvest losses and increased exposure to diseases (Mäkinen et al., 2018).
12	Heading-flowering	Maximum number of consecutive days with rainfall above 5 mm	Increased risk of diseases
13	Flowering-maturity	Maximum number of consecutive days with rainfall above 5 mm	Increased risk of diseases

The cold stress indicator counts the number of days when the minimum daily temperature (measured at the standard level of 2 m) drops below 2 °C (Table 3). Temperatures below this threshold, after the overwintering period, may increase the risk of permanent damage to stems, tillers and reduce the number of spikes. Heat stress indicators capture the number of hot days with maximum daily temperature above a pre-defined threshold (here set to 28 °C). These temperatures can cause partial or complete sterility of florets and a decline in photosynthetic rate, when occurring during the flowering period. During the grain filling period, they speed up the development, accelerate leaf senescence and lower yield biomass (Table 3). The frost and heat stress indicators can provide guidance on sowing dates selection, variety selection and irrigation planning (Table S1).

**Table 3:** Temperature related indicators characterizing conditions during different periods of winter wheat growth.

Indicator	Phenological stages	Description	Impacts on crop growth considered
14	Booting-flowering	Number of days with minimum temperature below 2 °C	Reduction of tiler and spike number, pollen sterility, stem damage (Frederiks et al., 2015)
15	Booting- end of flowering	Number of days with maximum daily temperature above 28 °C	Partial/complete sterility of florets, decline in photosynthetic rate (Akter and Rafiqul Islam, 2017, Porter and Semenov, 2005)
16	End of flowering – maturity	Number of days with maximum daily temperature above 28 °C	Exposure to heat speeds up development and decreases yield, decline in photosynthetic rate (Akter and Rafiqul Islam, 2017, Porter and Semenov, 2005)

Table 1, Table 2, Table 3 provide the full information and description of the proposed set of indicators here selected according to the growth phases of winter wheat (Fig. 1). To provide users a more flexible software, as well as to take into account cases when limited agronomic information is available, the temporal aggregation period for deriving the indicators can be selected either as static (i.e. calendar period when specified growing stages generally occur) or dynamic by using the implemented phenological model. As for the latter one, the temporal aggregation period is defined by *DVS* limits for each sensitive growing period and its inter-annual variability is temperature driven. For example, the flowering period might occur earlier (later) with respect to the fixed calendar period in case of warmer (cooler) preceding period.

#### 2.1.4 Quantifying the risk

Agro-climate indicators describe the risk associated with different types of weather and climate events that can cause losses of crop yield quantity and quality as well as increase the chance of disease breakout. However, the quantification of the risk levels is not always straightforward.

The intensity of drought, as estimated by the SPEI, is generally classified as follows: moderate (SPEI between −1.5 and −1), severe (SPEI between −2 and −1.5) and extreme (SPEI below −2). Positive SPEI values indicate wetter conditions with respect to the climatological norm: moderately wet (SPEI between 1 and 1.5), very wet (SPEI between 1.5 and 2) and extremely wet (SPEI above 2). Indicators of excessive wetness and cold and heat stress are based on the number of days when the event occurs. The duration of an event (number of days) indicates the associated risk, with higher number increasing the risk of negative impact on crop growth (i.e. more days with specified climate event will likely cause more relevant crop damages).

Thresholds may be objectively determined when sufficient impact local data are available, such as crop yield quality and quantity at harvest, occurrence of various diseases during different phases of crop growth and damages on plant tissue (e.g. leaves) due to frost and/or heat stress. This way the values of indicators may be directly used to quantify risk levels. To appreciate the importance of local data, a few examples can be used. When a crop is grown on sandy soils it will experience drought stress earlier than the same crop grown on clay soils with higher water retention capacities. Similarly, a wheat variety that is less resistant to heat stress might experience yield loss already during few days exposed to high temperatures, while more resistant varieties would be less responsive (Akter and Rafiqul Islam, 2017). We illustrate the risk-based evaluation for disease occurrence in section 3.1.

### 2.2 *Clisagri* as climate service

*Clisagri* has been co-designed with farmers and agronomists, who characterized weather and climate events during the wheat growing season that can potentially result in losses of crop yield quality and quantity, occurrence of diseases and weeds, problems related to grain storage after harvest and difficulties related to crop fertilization. During the co-design approach we translated their perceived harmful weather and climate events into a set of agro-climate indicators. Farmers also identified measures and actions to be taken to reduce the impacts of these events. These measures are described in terms of their technical value and potential applicability in Table S1. Given the co-design nature of *Clisagri* development, an important added value resides in the ease of interpretation of indicator values for farmers. Nevertheless, an intermediate agent may be necessary in some cases to convert the output into clear operative recommendations. Thus, *Clisagri* has already been integrated into a Decision Support System (DSS) developed by HORTA (https://www.horta-srl.it), a permanent platform for enhancing results from research in the agro-alimentary sector. This is highly relevant step in the information outreach, as the HORTA DSS has the capability to reach out hundreds of farmers in Italy and in other regions of the world.

*Clisagri* can be applied to climate observations of the past to get both risk evaluation and better process-understanding of impacts. When combined with seasonal-to-decadal climate predictions and climate projections, this tool can serve as a decision supporting system. A range of lead time scales can be used, from monthly and seasonal forecasts (up to a year) to decadal predictions (up to 10 years) and climate projections (up to the end of the century).

**Fig. 2:**
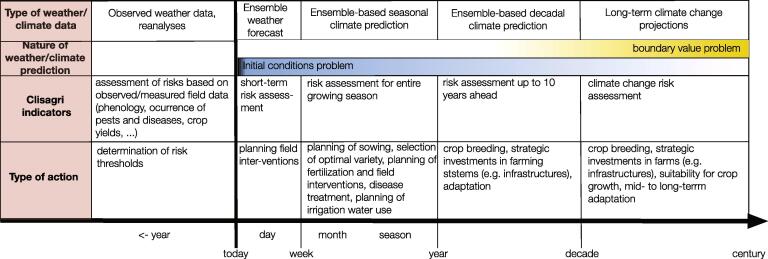
Applicability of *Clisagri* for risk assessment on different time scales, and actions that can be taken given the *Clisagri* output.

Fig. 2 shows the applicability of *Clisagri* on different time scales and explains the actions that may be taken. When considering the recent past, the *Clisagri* indicators can be used for the assessment of risks based on measured field data (e.g. crop yield quantity and quality and disease occurrence); this can provide an efficient method for the determination of specific thresholds for the selected indicators (see Section 3.1). Assessment of risk for the future is clearly probabilistic, given the nature of climate predictions and projections. Seasonal-to-decadal predictions based on climate models combine a boundary condition problem (simulating the response to forcings and the feedbacks between them) with an initial condition problem (depending on the current state of climate system, Doblas-Reyes et al., 2013). In such cases, an ensemble of near-future climate realisations must be used within a probabilistic framework to provide probability to expect certain climate conditions (e.g. being close to normal, above normal or below normal; Ceglar et al., 2018, and references therein). Section 3.2 provides an example of using seasonal climate predictions and climate projections with *Clisagri.*

Clearly, these different time scales are associated with different levels of decision making (e.g. Ceglar et al., 2018, and references therein). Short-term weather forecasts can be used to plan field operations, such as field preparation for sowing, ploughing, fertilization, slurry and harvesting. Seasonal forecasts, from one month to one year ahead, can provide strategic information for farmers, such as variety selection and fertilization requirements. While, predictions on decadal time scale could provide information on infrastructure investments (e.g. irrigation), crop breeding, and regional-to-national policy planning (Fig. 2). Table S1 provides more detailed information on the technical value and potential usability of *Clisagri* indicators, as identified by farmers and agronomists during the co-design process. *Clisagri* is designed to primarily provide climate information to help farmers making climate smart decisions. These include decisions that farmers make before, during and immediately after the crop growing season (Table S1). Before sowing, the tool can help answering questions such as: (i) is a more drought resistant variety for the next growing season needed? (ii) what is the optimal sowing date to avoid unfavourable weather conditions? During the growing season, the provided climate information: supports planning of field operations and selection of the optimal timing for fertilization; helps to reduce the negative effects of unfavourable/extreme weather and climate events during the sensitive growing stages (e.g. irrigation in the case of expected heat wave and/or drought events); supports controlling pests and diseases; supports the planning of the harvesting campaign.

Beyond the seasonal time scale, the tool can be used with decadal predictions and climate projections to, e.g., better characterize changes in occurrence and intensity of extreme events during the crop growth cycle, providing important information to plant breeders for the development of climate resilient crop varieties (Fig. 2).

## 3 Results

*Clisagri* allows users to calculate and visualize all the agro-climate indicators described in the Methods section. The development version of the package is available for download on GitHub (https://github.com/ec-jrc/Clisagri)*.* Table 4 provides a list of core functions available. The current approach supports the calculation of 16 indicators with either static or dynamic time periods (defined by crop development stage). Additionally, the graphical tool allows mapping intensity of climate events that could affect growth and yield of pre-identified crop varieties, as given by the combination of *TSUM1* and *TSUM2* (thermal requirements for vegetative and reproductive periods, respectively) for the standard approach or combination of *TSUMs* (characterizing each sub-phase) in the multi-phase phenological model.

**Table 4:** List of *Clisagri* core available functions.

Function	Description
clis.agri	Calculates a set of agro-climate indicators characterizing weather conditions during sensitive stages of durum wheat growth
phenology	Function calculating dynamic phenological development stages of wheat based on provided weather data and wheat variety related parameters characterizing thermal requirements for vegetative and reproductive growing periods
phenology.parameters	Description of phenological parameters used by phenology function
hazard.map	Displays 2D plot of occurrence of event based on provided range of TSUM1 and TSUM2
clim.plot	Displays time series plot of intensity of unfavourable events based on different combinations of TSUM1 and TSUM2
phenology.breeder	Function calculating dynamic phenological development stages of wheat based on provided weather data and wheat variety related parameters characterizing thermal requirements for 6 different sub-phases
breeder.calc	Fitness function providing fitness score for genetic optimization algorithm
breeder.plot	Displays phenological development stages of optimal variety, selected by genetic algorithm, and indicators that were used to minimize the impact on crop growth

Input climate data consists of minimum and maximum daily air temperature and daily precipitation cumulates. All the functions can run with observational as well as with predictions and projections (Fig. 2). COPERNICUS Climate Data Store (CDS, https://cds.climate.copernicus.eu/) can be considered as potential data source of climate predictions and projections.

### 3.1 Durum wheat production in the Mediterranean region: a case study

Durum wheat is mainly grown in the Mediterranean environment (Guzmán et al., 2016), and mainly used in the production of pasta (Nazco et al., 2014). The Mediterranean crop growing environment has become and will be challenging due to increasing temperatures, changes in precipitation regimes, and more frequent and intense extreme events, such as droughts and heat waves (Naumann et al., 2018, Zampieri et al., 2017, Fontana et al., 2015). By integrating seasonal climate predictions, *Clisagri* represents a useful tool to anticipate impacts and provide decision makers with timely information to support the agro-management process and the entire farming system. To demonstrate the current *Clisagri* functionality, we have selected durum wheat production areas in Italy.

#### 3.1.1 Risk assessment integrating impact data

To illustrate a risk evaluation based on *Clisagri*, we present here an estimation of risk for Septoria Complex occurrence between heading and maturity for durum wheat grown in three locations in Italy: Ravenna, Jesi and Foggia. The observations of disease severity were collected from controlled field experiments between 2014 and 2019, where durum wheat was not treated against the occurrence of disease. The dependency of disease severity is modelled using the number of days with rainfall above 5 mm as an indicator of climate impact (indicator 10 in *clis.agri*). A copula approach (Nelsen, 2006) is used to analyse the joint probability distribution between the climate indicator and the disease severity. Copula based approaches have recently gained attention in studies focusing on agricultural risks associated with adverse climate conditions (Ribeiro et al., 2020, and references therein), also due to their capability to model nonlinear dependency structure in data. Kernel estimator has been used here to fit the bivariate copula density (Nagler, 2018).

**Fig. 3:**
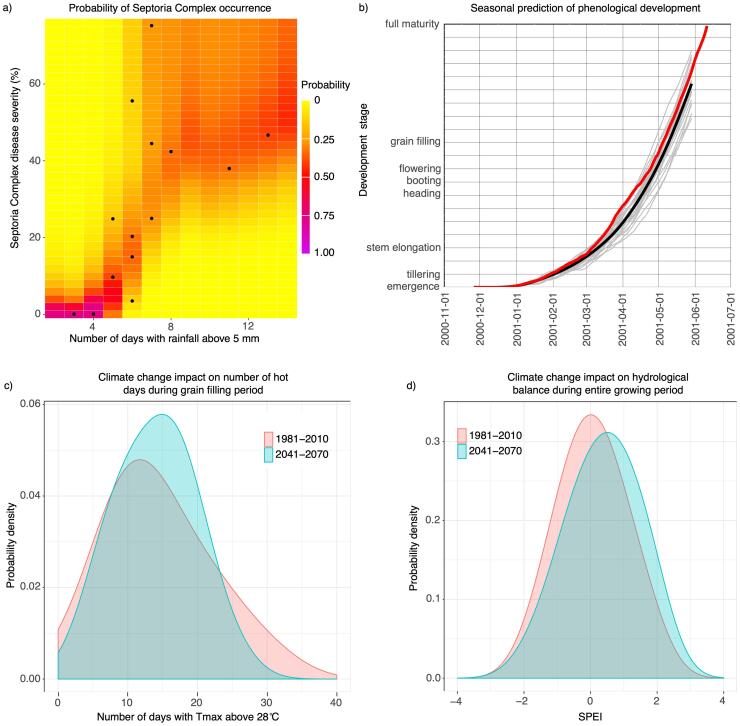
a) Modelled dependence between Septoria complex disease severity and number of days with rainfall above 5 mm between heading and maturity for durum wheat grown in three Italian locations: Ravenna, Jesi and Foggia. Colours denote the probability of observed disease severity for different number of days with rainfall amount above 5 mm. Dots represent the observational pairs based on disease severity from the field experiments and agro-climate indicator calculated on measured rainfall data. b) Seasonal prediction of phenological development, initiated in November 2000, for durum wheat grown in Ravenna. Grey lines represent simulations based on 25 different ensemble members, and the thick black line represents the ensemble mean. Red line represents the simulated phenological development based on observed climate data. c) Probability density of the number of hot days during the grain filling period (durum wheat grown in Ravenna) for the reference period (1981–2010) and mid of 21st century (2041–2070). No adaptation measures were assumed in the simulations (i.e. the same sowing date, TSUM1 and TSUM2 were used for both periods). d) Same as c, but for hydrological balance during entire growing period of durum wheat (i.e. between sowing and maturity).

Fig. 3a shows the dependency structure and probability of Septoria Complex severity for different number of days with rainfall amount above 5 mm. The probability of disease development is low if less than 5 wet days occur between heading and maturity. The severity of disease sharply increases if more than 5 such days are recorded, as shown by modelled bivariate copula density. The latter remains high in upper right quadrant of the graph, indicating that more than 8 wet days would likely cause the disease severity above 40%. This example illustrates that for durum wheat grown in the three locations, a threshold of 5 days for selected agro-climate indicator can be used to indicate high risk of severe of Septoria Complex occurrence.

#### 3.1.2 Using *Clisagri* with climate predictions and projections

The entire set of agro-climate indicators can be calculated using both seasonal-to-decadal climate predictions and climate projections. This type of climate information is usually provided in terms of ensembles, reflecting the uncertainty in initial conditions of the climate system, climate model structure and/or boundary conditions. This data can be used with *Clisagri* to estimate the phenological development and the risk associated with unfavourable/extreme events in the future. Fig. 2b illustrates the use of seasonal climate forecast to predict the phenological development of durum wheat variety grown in Ravenna. Seasonal predictions (from the ECMWF SEAS5 system**;** Johnson et al., 2019) were obtained from the COPERNICUS Climate Data Store. These predictions, belonging to a hindcast experiment, were initiated for November 2000 and consist of an ensemble of 25 different realizations of climate conditions (reflecting uncertainty in initial conditions) for the lead time of 6 months. The seasonal prediction run, initiated in November each year, can provide beneficial information to farmers as it becomes available around sowing time and covers large part of the growing season ahead of time, thus providing predicted climate conditions for the sensitive wheat growing stages.

The phenological development of durum wheat can be simulated by using the *phenology* function (Eq. (1)) of *Clisagri*, which requires daily meteorological data (daily minimum and maximum temperatures), sowing dates and a parameter matrix. The parameter description for phenological model can be invoked by calling the function *phenology.parameters()*. These parameters should reflect the wheat variety under consideration, since they are used to simulate daily development rate, vernalization and photoperiod sensitivity. The default set of parameters represents a typical Mediterranean durum wheat variety grown in Italy. The most relevant parameters are *TSUM1* and *TSUM2* representing, respectively, the thermal requirement to reach flowering and physiological maturity. The function *phenology* can be used to simulate DVS for unique combination of *TSUM1* and *TSUM2*, as provided in the parameters matrix.

When predicted climate data is provided as an ensemble of different realizations, phenological development is calculated for each ensemble member separately, thus giving users the possibility to summarise the ensemble of phenological development predictions. Fig. 2b illustrates phenological development prediction for wheat variety grown in Ravenna sown towards the end of November 2000. Shown are the ensemble mean and each ensemble member, giving an overall overview of the ensemble spread and the uncertainty related to the phenological prediction. The phenological development simulated using observed climate data (red curve) falls within the range of the ensemble spread. These simulations can further be used to calculate the agro-climate indicators (not shown here).

Fig. 3cd illustrates another example based on climate projections to estimate changes in the number of hot days during the projected grain filling period together with changes in the hydrological balance during the entire wheat growing period. To perform such an example, we have used an ensemble of five different climate model projections for Ravenna from the high-resolution and bias adjusted EURO-CORDEX simulations (Dosio, 2016) under the RCP8.5 scenario (a detailed description of these models can be found in Ceglar et al. 2019a). Fig. 3c shows the estimated probability density functions of the number of hot days during the grain filling for the reference period (1981–2010) and mid of 21st century (2041–2070). The probability of having between 10 and 20 hot days substantially increases in the future, while the probability of having more than 30 days practically diminishes. This can be explained by considering that the grain filling period in the future is projected to shorten (not shown) due to the increased mean temperature. As for this specific example, the thermal requirements TSUM1 and TSUM2 as well as the sowing date have been assumed to remain the same of the control run (i.e. no adaptation). This example clearly shows that with no adaptation the grain filling will shorten, and at the same time crop would be exposed to higher risk of heat stress. As for the hydrological balance for the entire growing season (Fig. 3d), the probability of having slightly wetter conditions will increase with climate change, mainly due to the shorter growing season and the projected increase in winter precipitation.

### 3.2 Variety selection

**Fig. 4:**
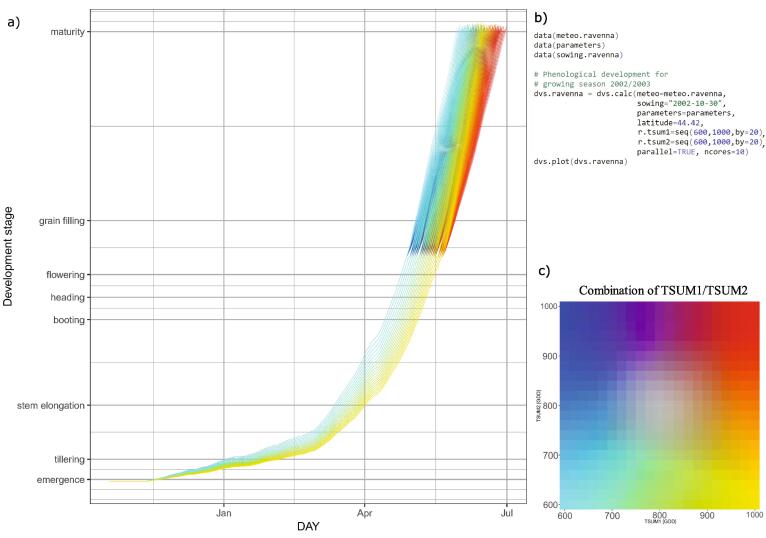
Simulated durum wheat development stage for the crop growing season 2002/3 in Ravenna (a) based on different combinations of thermal requirements for vegetative (*TSUM1*) and reproductive (*TSUM2*) growing periods. To distinguish between different combinations, the color of each DVS time series in (a) is related to scale in (c), with each color representing a unique combination of *TSUM1/TSUM2*. The R code to produce the DVS time series graph is shown in (b).

The observed meteorological data used in this section were obtained from the EC-JRC MarsMet, established and maintained for the purpose of crop growth monitoring and forecasting (Toreti et al., 2019b). Depending on the climate of the region under analysis, different combinations of *TSUM1* and *TSUM2* may lead to different exposure of wheat to unfavourable/extreme weather and climate events during the growing season. To assess the spectrum of varieties with different thermal requirements, *Clisagri* provides a function to explore wide ranges of *TSUM1/TSUM2* combinations (*dvs.calc*). The possible range of *TSUM1* and *TSUM2* values, here shown, has been determined based on the calibrated values for different wheat varieties in southern Europe and the Mediterranean regions (Ceglar et al., 2019b) and the expert opinion from HORTA agronomists. To demonstrate how this function works, we focus on the 2002/3 growing season late spring and early summer, which usually coincide with flowering and grain filling period of durum wheat. Exceptionally warm and dry conditions were observed in 2003 in Italy (e.g. Fontana et al., 2015). Fig. 4 shows the simulated development stage in Ravenna (assuming the sowing date being at the end of October 2002), using different combinations of *TSUM1* and *TSUM2* (represented by different colors in Fig. 4c). Assuming the base temperature of 0 °C, the range of *TSUM1* and *TSUM2* between 600 and 1000 growing degree days (GDD) is typical for Mediterranean durum wheat varieties grown in Italy (Isidro et al., 2011). Fig. 4a demonstrates that the timing of different development stages can vary considerably depending on the selected variety. For example, flowering occurrence can range between mid-April and the beginning of May, depending on *TSUM1*. Different combinations of *TSUM1/TSUM2* give many more possibilities on the development after flowering occurs (i.e. for each *TSUM1* there is an entire range of *TSUM2* values defining the thermal length of the reproductive period). The varieties with the longest thermal requirement would have reached maturity until the end of June in 2003.

**Fig. 5:**
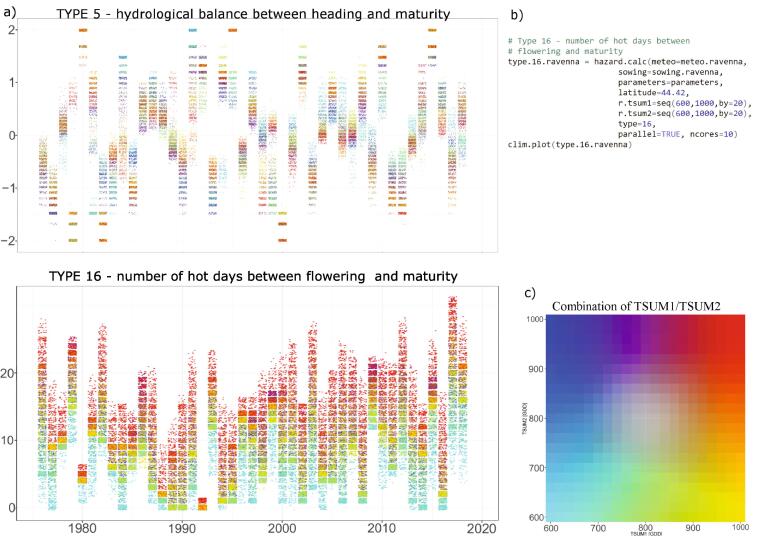
Simulated hydrological balance between heading and maturity (a) and number of hot days between flowering and maturity, based on different combinations of *TSUM1/TSUM2* (indicated by different colors). To distinguish between different combinations, the color of each simulated indicator is related to scale in (c), with each color representing a unique combination of *TSUM1/TSUM2*. The R code to produce the indicator of type 16 (number of hot days) is shown in (b).

To further illustrate the climate effects during the different growing stages, we calculate the hydrological balance and the number of hot days between the beginning of flowering and maturity (indicators 5 and 16, respectively). Choosing different combinations of *TSUM1/TSUM2* clearly results in a wide range of drought and heat stress conditions that crop could experience during and after the flowering (Fig. 5).

In most of the observed years, drought can be avoided by selecting an optimal subsample of *TSUM1/TSUM2* combinations (Fig. 5a). For example, drought in 2000 could have been avoided with varieties having earlier flowering (i.e. *TSUM1* roughly below 800 GDD). Other combinations of thermal requirements result in growing conditions affected by drought. Interesting conditions characterize the years 2000 and 2015, when all possible combinations of *TSUM1/TSUM2* result, respectively, in drought and wet conditions.

As for the number of hot days (Fig. 5a), higher thermal requirements generally result in higher exposure to heat stress. This is expected as the probability of exposure to high temperatures increases with later flowering and longer grain filling period. Nevertheless, a range of thermal requirements can be selected in order to reach an optimal balance between heat-exposure and length of the grain filling period (needed to reach adequate crop yield). Heat stress exposure varies substantially according to the selected combinations of *TSUM1/TSUM2*. When late spring and early summer only experience few hot days, the selection of thermal requirements combination becomes less relevant in terms of exposure to heat stress (e.g. in 1980 and 1992). While it can become crucial in years such as the 2017, characterized by exposure to heat stress spanning from 5 to 31 days depending on thermal requirements. Therefore, a time-dependent climate informed variety selection to be sown may ensure an optimal compromise in terms of exposure and minimum length of the grain filling period to reach sufficient biomass accumulation. If varieties with early flowering need to be sown for specific reasons, then additional assessments should be made to evaluate the exposure to cold temperatures between booting and flowering (indicator 14, not shown here).

**Fig. 6:**
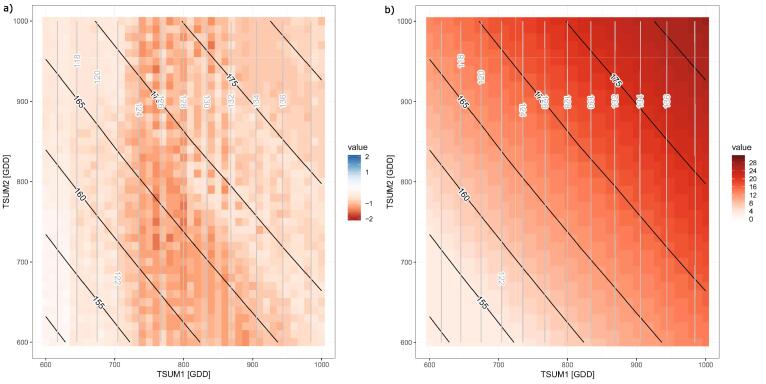
Exposure to drought between heading and maturity (a) and heat stress between flowering and maturity (b) during the growing season 2002/3 in Ravenna, depending on various combinations of variety thermal requirements (TSUM1 and TSUM2). Values below −1 in (a) indicate drought conditions. Values in (b) represent the number of hot days. Grey (black) lines represent the isolines of simulated flowering (maturity) date.

Fig. 6 shows how different thermal requirements lead to varying exposure to drought and heat stress during the 2002/3 crop growing season in Ravenna. Varieties with shorter growth cycle (i.e. *TSUM1* and *TSUM2* below 700 GDD) could have been used to avoid intense drought and heat stress. Maturity in these varieties would have been reached at the beginning of June 2003, thus largely avoiding intense heat stress occurring later in the season. On the contrary, varieties with longer thermal requirements (*TSUM1* and *TSUM2* above 700 GDD) are severely exposed to drought and heat stress.

Often, farmers also adapt by changing sowing dates. This factor can be also considered in *Clisagri* optimization strategy, by perturbing the associated value in the parameters matrix. Users can either repeat the procedure for optimal TSUM1/TSUM2 combinations under different sowing dates or run the *clisagri* function assuming constant TSUM1/TSUM2 (i.e. representing currently grown variety) and perturb only sowing dates. By comparing the values of the selected agro-climate indicators, users can then decide upon sowing date that minimises the risk of selected unfavourable/extreme weather and climate events.

Perturbing *TSUM1* and *TSUM2* values in the standard approach affects all sub-phases of wheat development proportionally (i.e. increasing *TSUM1* results in increased length of all sub-phases from sowing to flowering). The multi-phase model addresses this shortcoming by simulating phenological development for six sub-phases, using a set of six *TSUMs* and *T_b_* parameters for each sub-phase. Typical parameter values for durum wheat varieties grown in Italy are shown in Table 5. The parameter description for the multi-phase model can be invoked by calling function *phenology.parameters(breeder = TRUE).*

**Table 5:** Parameter values (representing typical Italian durum wheat varieties) for multi-phase phenological model.

Phase	TSUM range (GDD)	Tb (°C)
sowing – emergence	120–200	2
emergence – tillering	35–140	−2
tillering – stem elongation	100–220	−2
stem elongation – booting	100–200	0
booting – flowering	50–150	0
flowering – ripening	450–550	9

A systematic search of the optimal variety (as in the case of the base phenological model) in this case becomes computationally highly intensive as $\prod_{i=1:6}n_{i}$ possible combinations ($n_{i}$ representing the number of TSUM discretization classes for each sub-phase) must be analysed. We have therefore implemented an optimization method based on genetic algorithm (GA; Scrucca, 2013). In short, a genetic algorithm is a stochastic search algorithm which reflects the process of natural selection where the fittest individuals are selected for reproduction.

Five phases are important for the genetic algorithm: initial population, fitness function, selection, crossover and mutation (Scrucca, 2013). The fitness function determines the performance of the individuals, i.e. their ‘score’, thus the probability to be selected in the subsequent generation of individuals. *Clisagri* provides a fitness function (*breeder.calc*) that can incorporate all the agro- climate indicators. The main idea behind is to maximize the fitness score by bringing selected agro-climate indicators to a value which minimizes the impact on the crop growth given the climate conditions during the growing season. The selection phase identifies the fittest individuals and passes them to the next generation. Crossover forms offspring from two parents by combining part of the genetic information from each; while, the mutation randomly alters the values of genes in a parent chromosome, thereby maintaining diversity within the population and preventing premature convergence and thus local optimization. In simplified terms, the *breeder.calc* function aims to find the best combination of thermal requirements for each wheat sub-phase that minimizes the unfavourable/extreme weather and climate events during the critical growth stages.

**Fig. 7:**
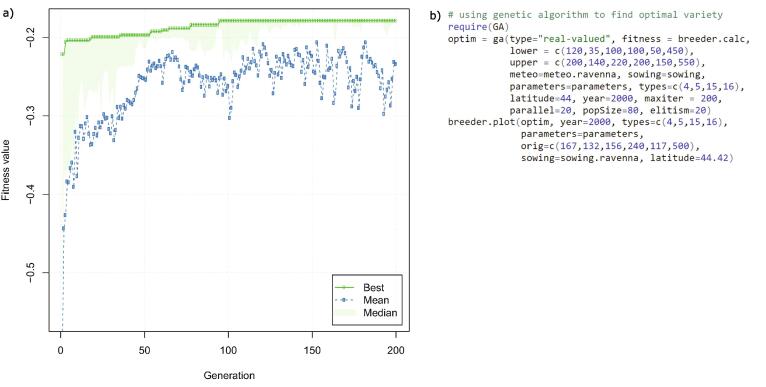
a) Best, mean and median fitness score values at each GA generation step. b) The R code to perform the optimal variety selection.

We present here an example of searching for the optimal set of sub-phase thermal requirements for the durum wheat growing season 1999/2000 in Ravenna. We optimize the variety selection on the climate impacts during the pre-flowering and grain filling phases using indicators of hydrological balance and heat stress (types 4, 5, 15 and 16; see Fig. 1). The GA converges towards a solution where ideally the selected wheat variety would experience neither drought nor wet conditions (i.e. values of hydrological indicator between −1 and 1) and it would be exposed to hot weather for the shortest possible period. The lower and upper boundaries for thermal requirements in each sub-phase (Fig. 7a) are given in Table 5.

Fig. 7a represents the fitness score based on 200 iterations of the implemented GA. It takes roughly 100 iterations for the algorithm to converge towards quasi-stable solution, with fitness score moving between −0.3 and −0.2. The resulting thermal requirements maximizing the fitness score are as follows for each sub-phase (in GDD): 139 (PH1), 77 (PH2), 148 (PH3), 125 (PH4), 51 (PH5) and 453 (PH6).

**Fig. 8:**
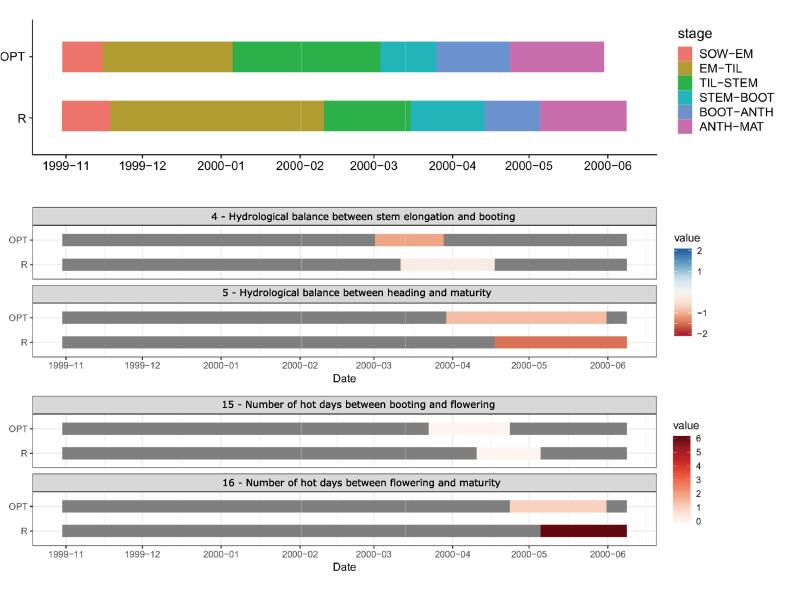
Phenological development stages of optimal (OPT) and reference (R) varieties for the growing season 1999/2000 in Ravenna along with the four agro-meteorological indicators selected for the GA-optimization. The values of the indicators are presented for both the optimal and the reference variety.

Fig. 8 represents each phenological sub-phase (PH1-PH6) of the variety having GA-optimized values of thermal requirements (OPT), obtained using the *breeder.plot* function. For comparison purposes, the growing season is presented also for a reference variety (R), typically grown in Emilia-Romagna during the ‘90 s (Rossi et al., 1997). Along with the phenological development, Fig. 6 also shows the agro-meteorological indicators that were used in the GA-optimization procedure. The most notable difference is in the length of the growing season, which is altogether slightly shorter for OPT. The variety selection favours shorter tillering and longer stem elongation period, when compared to the reference variety. Earlier occurrence of flowering and maturity in the optimal variety results in a considerable reduction of heat stress exposure during the grain filling period and also in avoiding drought conditions between heading and maturity.

Variety optimisation function *breeder.calc* can also be used with climate predictions and projections. At the decadal time scale, it may support crop breeders. When seasonal climate predictions are used, the *breeder.calc* considers all ensemble members equally when searching for optimal variety for the coming growing season. In this case, the optimization targets farmers who need to decide on the type of variety and plan the field interventions.

## 4 Future extensions

Below is the list of additional functionalities already planned to be integrated in the *Clisagri.*.

(i) Skill assessment of seasonal-to-decadal climate predictions. Even though the skill of seasonal weather forecast has been shown useful for European agriculture (Ceglar et al., 2018, Falloon et al., 2018), the integration of such products into *Clisagri* still requires spatio-temporal assessments. These should provide users with the complete information on seasonal forecast of the agro-climate indicators on the predicted phenological phases together with score indicators on the usefulness of these forecasts (i.e. on the added value in seasonal forecasts with respect to the climatological information available from observations).
(ii) Direct connection to COPERNICUS CDS and downloading of the relevant climate data for selected locations.
(iii) Additional crop types and agro-climate indicators.
(iv) Calibration of the phenological model with user’s field data.
(v) Additional phenological models providing users the possibility to select the preferred model.
(vi) Integration of dedicated sowing model, aiming to select optimal sowing date based on preceding weather conditions.

## 5 Conclusions

*Clisagri* provides user-oriented co-designed climate service tools for the agricultural sector. It makes available dynamic phenological models and offers an easy way to derive key agro-climate indicators to assess risks and exposure of crops. A full range of possible crop varieties can be used and tested, and it is already offered for wheat in the current version of *Clisagri*. It also allows users to analyse immediately the impact of past, current and future climate conditions on crop growth. Climate predictions and projections can also be used although some pre-processing steps are not available yet (i.e. skill assessment, bias-adjustment).

The added value of *Clisagri* as sectoral climate service tool has been clearly demonstrated for durum wheat grown in Italy. *Clisagri* provides farmers with climate information to support decisions at different stages before and during the crop growing season.

Finally, as *Clisagri* is open source and freely available we encourage users to run it and report any issue and ideas on future extensions.

## CRediT authorship contribution statement

**A. Ceglar:** Conceptualization, Formal analysis, Software, Methodology, Writing - original draft, Writing - review & editing. **A. Toreti:** Conceptualization, Methodology, Writing - review & editing. **M. Zampieri:** Methodology, Writing - review & editing. **V. Manstretta:** Methodology, Writing - review & editing. **T. Bettati:** Software, Methodology, Writing - review & editing. **M. Bratu:** Software, Writing - review & editing.

## Declaration of Competing Interest

The authors declare that they have no known competing financial interests or personal relationships that could have appeared to influence the work reported in this paper.
