# Supplementary material for: Clisagri: An R package for agro-climate services
Source: Clim Serv. 2020 Dec;20:100197. doi: 10.1016/j.cliser.2020.100197 (PMC7789046; doi:10.1016/j.cliser.2020.100197)
Supplement: Supplementary data 1 [file mmc1.pdf]

Supplementary material for: Clisagri: An R package for agro-climate services

Authors: A. Ceglar[1,*], A. Toreti[1], M. Zampieri[1], V. Manstretta[2], T. Bettati[2], M. Bratu[1]

[1]European Commission, Joint Research Centre, via Enrico Fermi 2749, 21027 Ispra, Italy

[2]HORTA, Via Egidio Gorra 55, 29122 Piacenza, Italy

[*]Corresponding author, andrej.ceglar@ec.europa.eu

Table S1: The technical value and potential usability of the agro-climatic indicators provided in the Clisagri package, as perceived by farmers and agronomists during the indicator co-design process.

ID	Description	Technical value	Potential usability
1	Hydrological balance during pre-sowing period	The index can give a support for the decisions on soil tillage at sowing (ploughing vs. no till), on the sowing technique to be applied in the field (conventional vs. direct sowing), and on the application of fertilisers in the pre-sowing period (yes/no). In case drought conditions are foreseen, it is agronomically convenient to opt for no tillage or the application of conservation agriculture techniques, direct sowing and no application of fertilisers before sowing. In case wet conditions are foreseen, it is agronomically convenient to opt for conventional soil tillage and sowing, and to apply fertilisers in the pre-sowing period.	The application of the most suitable technique in the pre-sowing period can have an impact on the crop yield, and on the economic balance in the cultivation phase. The possibility to have seasonal predictions for this index can help the farmer in planning the field operations in advance. Predictions for future years (decadal predictions) can inform the decisions on the machineries to be purchased, considering that the equipment is different from conservation agriculture to conventional one.
2	Hydrological balance between sowing and emergence	Similar technical value as for indicator 1, when it comes to planning of field preparation and variety selection.<br><br>Additionally, in areas affected with the wheat mosaic virus, which is a soil borne disease transmitted by the plasmodiophorid *Polymyxa graminis*, this index can support wheat variety choice, as wet conditions at wheat emergence favour the infection.	Similar potential usability as for indicator 1.<br><br>Additionally, in areas where the mosaic virus poses a problem this index can inform the choice of the wheat variety to be sown, indicating the need to opt for a resistant variety.

3	Hydrological balance during tillering	This index can give support for the application of nitrogen fertilisers, which is a critical decision at this stage. In case of cold and rainy winters, the crop will benefit from a higher amount of fertilisers, while it is important not to exceed in nitrogen fertilisation in case winter conditions were mild and dry.	The application of the optimal amount of fertiliser in the tillering phase can have an impact of the yield of the crop. The possibility to have seasonal predictions for this index can help the farmer in planning the field operations in advance, and in the choice of the most suitable form of fertilisers (slow release products, or products with inhibitors to minimise fertilisers losses).
4	Hydrological balance between beginning of stem elongation period and end of booting	The index can give support for the application of nitrogen fertilisers, in the period when the crop has the higher demand for nutrients. Information can also be important for the crop protection from diseases, and for the eventual application of irrigation in case of drought.	The application of the correct amount of fertilisers, the optimal crop protection strategy and the eventual irrigation (in case of drought) have direct impact on grain yield and quality. The possibility to have seasonal predictions for this index can help the farmer in planning the field operations in advance, and in the choice of the most suitable form of fertilisers (slow release products, or products with inhibitors to minimise fertilisers losses). The possibility to know in advance the foreseen disease pressure can guide the farmer in the choice of particular plant protection products, i.e. on the base of their efficacy.
5	Hydrological balance between beginning of heading and full maturity	This index can give a support for the preparation of the crop protection measures against diseases, in particular for *Fusarium Head Blight* (FHB). FHB is caused by a complex of fungal species of the genus *Fusarium*, which infect wheat heads at flowering and can cause both a decrease in grain yield and quality, and the grain contamination from mycotoxins.	Given the seasonal prediction of this index before the sowing, can help the farmer to select the variety that is more drought resistant (in case the seasonal prediction foresees drought).<br><br>Additionally, the application of the optimal crop protection strategy have direct impact on grain yield and quality. The possibility to have seasonal predictions for this index can help the farmer in planning the field operations in advance, including irrigation in the case of drought, and in the choice of the most suitable crop protection products, i.e. on the base of their efficacy.

6	Hydrological balance between sowing and full maturity	The index can support the decision on: i) soil tillage and sowing technique, ii) fertiliser application, iii) crop protection strategy; iv) need for irrigation, which all together contribute to yield and quality of grains at harvest.	The application of the most suitable tillage and sowing techniques, the correct amount of fertilisers, the optimal crop protection strategy and the eventual irrigation (in case of need) have direct impact on grain yield and quality. The possibility to have seasonal predictions for this index can help the farmer in planning the field operations in advance, and in the choice of the most suitable machinery and equipment, technical inputs such as fertilisers and plant protection products. Predictions for future years (decadal predictions) can inform the decisions on the machineries to be purchased.
7	Rainfall amount during pre-sowing period	This index provides absolute amount of rainfall during pre-sowing period. As such it provides complementary information to index 1, which represents a hydrological balance (i.e. it also includes the evapotranspiration component). The technical value for this index is similar to index 1.	The potential usability for this index is similar to index 1.
8	Number of rainy days with rain above 10 mm during tillering period	The index can give a support for the application of nitrogen fertiliser, as certain amount of rainy days is need for the fertiliser activation. The index is also relevant for the application of chemical for weed management, which also require rain to be activated in the soil. The index can also give information on the possibility to apply legumes intercropping in wheat fields. This technique consists in the sowing of legumes in the wheat inter-row at wheat tillering, and benefits from harrowing after the distribution of legumes' seeds.	The application of the optimal amount of fertiliser in the tillering phase can have an impact of the yield of the crop. The index can help the identification of the best time to perform fertilisation, application of chemical for weeding, and the sowing of intercrops in the wheat field.The possibility to have forecasts for this index can help the farmer in planning the field operations in advance, in the most suitable conditions and with the most suitable products.
9	Number of days with rain above 40 mm during tillering	This index is complementary to index 8 and gives a support for the application of nitrogen fertilisers, which is a critical decision at this stage. In case heavy rain, there is a higher risk of leaching.	Similar to index 8.

10	Number of days with rain above 5 mm between heading and full maturity	The index can give support for the decisions on crop protection from diseases, in particular for *Fusarium Head Blight* (FHB). FHB is caused by a complex of fungal species of the genus *Fusarium*, which infect wheat heads at flowering and can cause both a decrease in grain yield and quality, and the grain contamination from mycotoxins.	The application of the optimal crop protection strategy has a direct impact on grain yield and quality. The possibility to have seasonal prediction for this index can help the farmer in planning the field operations in advance, and in the choice of the most suitable crop protection products, i.e. on the base of their efficacy.
11	Number of days with rain above 40 mm between beginning of heading and full maturity	The index can give an indication on the expected yield and quality of grain at harvest. In the absence of heavy rain during this period, quality of harvested grain will probably be high (protein content), while in case of heavy rain events there is an increased risk for quality decrease (fungal infections, black point problems and/or mycotoxin contamination.	The possibility to have seasonal prediction for this index can help elevators to plan in advance the most suitable storage strategy for grain lots with different qualitative characteristics.
12	Maximum number of consecutive days with rain above 5 mm between heading and flowering	This index is a complementary to the information given by index 10. As such, it can support the decisions on the crop protection from diseases, in particular for FHB.	Similar to index 10.
13	Maximum number of consecutive days with rain above 5 mm between flowering and full maturity	This index is a complementary to the information given by index 11. As such, it can provide a risk measure for grain quality at harvest.	Similar to index 11.
14	Number of days with minimum daily temperature below 2 °C between booting and flowering	The index can give an indication on the frost risk and related damage to the plants, leading to a reduction of grain yield.	The possibility to have seasonal prediction for this index can give the farmer an indication on the expected yield.
15	Number of hot days with maximum daily temperature above 28 °C between stem elongation and flowering	The index can give support the decision on the irrigation, which can help counterbalancing the effect of the heat stress, with positive effect on yield.	The seasonal prediction for this index gives farmers the possibility to plan the irrigation, and gives them an indication on the expected yield amount and quality. Additionally, having predictions early enough in season (sowing period) can help farmers to adapt sowing dates and select optimal variety to avoid the heat stress during the most sensitive growth stages.

16	Number of hot days with maximum daily temperature above 28 °C during grain filling period	This index can give an indication on the possibility to have heat stress for the plants, impacting on grain yield and quality.	Similar to index 15.
